# Constructing a fish metabolic network model

**DOI:** 10.1186/gb-2010-11-11-r115

**Published:** 2010-11-29

**Authors:** Shuzhao Li, Alexander Pozhitkov, Rachel A Ryan, Charles S Manning, Nancy Brown-Peterson, Marius Brouwer

**Affiliations:** 1 Gulf Coast Research Laboratory, Department of Coastal Sciences, University of Southern Mississippi, 703 East Beach Drive, Ocean Springs, MS 39564, USA; 2 Current address: Emory Vaccine Center, 954 Gatewood Rd, Atlanta, GA 30329, USA; 3 Current address: Max Planck Institute, August-Thienemann-Str. 2, Ploen 24306, Germany

## Abstract

We report the construction of a genome-wide fish metabolic network model, MetaFishNet, and its application to analyzing high throughput gene expression data. This model is a stepping stone to broader applications of fish systems biology, for example by guiding study design through comparison with human metabolism and the integration of multiple data types. MetaFishNet resources, including a pathway enrichment analysis tool, are accessible at http://metafishnet.appspot.com.

## Rationale

Small fish species are widely used in ecological and pharmaceutical toxicology, developmental biology and genetics, evolutionary biology and as human disease models. Among the species commonly found in scientific literature are zebrafish (*Danio rerio*), medaka (*Oryzias latipes*), stickleback (*Gasterosteus aculeatus*), European flounder (*Platichthys flesus*), channel catfish (*Ictalurus punctatus*), sheepshead minnow (*Cyprinodon variegatus*), mummichog (*Fundulus heteroclitus*), Atlantic salmon (*Salmo salar*), common carp (*Cyprinus carpio*), rainbow trout (*Oncorhynchus mykiss*) and swordtail (*Xiphophorus hellerii*). Each of these fish species has its own niche as a research tool. For example, *Xiphophorus *is a classic genetic model of melanomas [1,2], whereas medaka is a good model for reproductive and ecotoxicological studies [3]. Zebrafish, in particular, has risen to stardom in recent years, with a large collection of mutants and established techniques for transgenesis, expression studies, forward and reverse genetics and *in vivo *imaging [4-8]. The use of zebrafish as human disease models has also spiked significant interests [9-11]. Since small fish are currently the only vertebrate species that can be studied in high throughput, their future in modern biomedical sciences is brighter than ever [12,13].

Fish genomics is also taking off. Thus far, whole genome sequences are available for five fish species:

*D. rerio*, *O. latipes*, *T. rubripes*, *T. nigroviridis *and *G. aculeatus*. DNA microarrays have been applied to study gene expression in many more fish species [14-18]. However, fish functional genomics is far behind other model organisms. In the example of sheepshead minnows, which are used in our lab for ecotoxicology, gene annotation is poor and no pathway analysis tool is readily available for interpreting DNA microarray data. The situation is similar for other fish species, with zebrafish perhaps an arguable exception. Bioinformatic tools that fill in this gap in fish functional genomics are highly desirable [17]. Oberhardt *et al*. [19] summarized the five applications of genome-wide metabolic network models: '(1) contextualization of high-throughput data, (2) guidance of metabolic engineering, (3) directing hypothesis-driven discovery, (4) interrogation of multi-species relationships, and (5) network property discovery.' While significant interest exists for a fish metabolic network model in all five categories, the immediate and primary application of our model will be the interpretation of high throughput expression data, especially pathway analysis, which can be done either by direct mapping to metabolic genes [20,21] or via established enrichment statistics [22,23]. This model will also provide a first glance of how fish metabolism resembles human metabolism, which should be instructional for the use of fish in many research areas [24]. This proposed first generation model will serve as a reference and stepping stone to further systems investigations, helping study design and hypotheses generation. As more data become available in the future, the model can be further refined to support broader applications.

The recent completion of genome sequencing of five fish species has paved the way for constructing a genome-wide fish metabolic network model. That is, all metabolic enzymes can be identified from complete genomes by sequence analysis, compounds can then be associated with enzymatic activities and a metabolic network can be constructed by linking these compounds and enzymes. This type of *ab initio *construction of metabolic networks has been carried out for many unicellular organisms [19,25-30].

However, *ab initio *construction alone is not yet feasible for vertebrate metabolic networks due to their complexity. Two high-quality human metabolic network models [20,31] have been published recently. Both studies included intensive human curation and comprehensive supporting evidence, including data from model species other than human. Thus, these two 'human' models can provide critical references for constructing a genome-wide fish metabolic network model, to help overcome the limitation of *ab initio *construction. Combining the integration of existing models and *ab initio *construction from whole genomes has been the strategy for our project. A metabolic model for zebrafish exists in the KEGG database [32].

However, our genome-wide model offers a significant expansion of the KEGG zebrafish model.

We will first report the construction process of this fish metabolic network model (MetaFishNet). We then use MetaFishNet to methodically comparefish and human metabolism to identify the most and least conserved pathways. The last sections of this paper will demonstrate the application of MetaFishNet in analyzing two sets of DNA microarray data: one from zebrafish as liver cancer model in public repository, the other from sheepshead minnow exposed to cadmium in our lab.

## Results and discussion

### Construction of MetaFishNet

**Figure 1:**
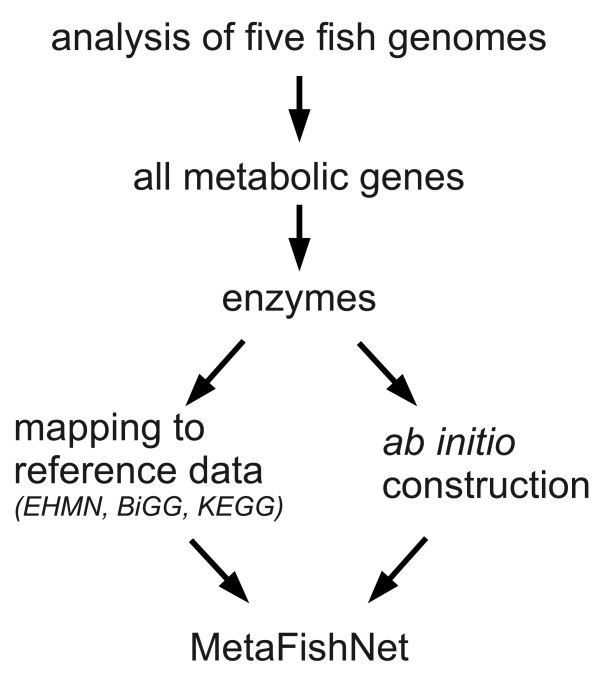
**Construction strategy of MetaFishNet**. See text for details.

Our genome-wide fish metabolic network, MetaFishNet, adopts a conventional bipartite network structure, where enzymes and compounds are two types of nodes. The construction strategy of MetaFishNet is shown in Figure 1. Details are given in the 'Method' section and Additional file 1, while a short description follows here.

We first analyzed all cDNA sequences from five fish genomes (*D. rerio*, *O. latipes*, *T. rubripes*, *T. nigroviridis *and *G. aculeatus*) to create a list of all fish metabolic genes via gene ontology. From this metabolic gene list, the corresponding enzymes were identified using either orthologous relationships to human genes or similarity to consensus enzyme sequences (Table 1). Two types of metabolic reactions are included in MetaFishNet. The majority consists of reactions in reference models that can be associated with fish enzymes. The rest of the reactions were created according to relationships between inferred enzymatic activity and compounds. The reference reactions in this project are data integrated from Edinburgh Human Metabolic Network (EHMN) [31], the human metabolic network from Palsson's group at UCSD (BiGG) [20] and the zebrafish metabolic network from KEGG. Finally, the whole network is formed by linking all reactions.

**Table 1:** Metabolic Enzymes found in five fish genomes

Species	Number of metabolic genes	Number of ECs
Zebrafish	3,853	654
Medaka	3,998	765
Takifugu	4,103	771
Tetraodon	4,424	782
Stickleback	4,324	791

To illustrate the construction process, let us consider two pieces of sequences from the medaka genome.

Sequence ENSORLG00000001750 is mapped to a human homolog PIK3CG, which is a phosphoinositide-3-kinase (enzyme commission number 2.7.1.153). This enzyme is associated to a reaction in the EHMN model that converts 1-Phosphatidyl-D-myo-inositol 4,5-bisphosphate to Phosphatidylinositol-3,4,5-trisphosphate. Thus, this same reaction is carried over to the MetaFishNet model. Another sequence ENSORLG00000018911 also has a human homolog, PIP4K2B, which is a phosphatidylinositol-5-phosphate 4-kinase with enzyme commission number 2.7.1.149. Although no reaction for this enzyme is found for any of the reference models, we learn from the KEGG LIGAND database that this enzyme converts 1-Phosphatidyl-1D-myo-inositol 5-phosphate to 1-Phosphatidyl-D-myo-inositol 4,5-bisphosphate. This reaction is added to MetaFishNet as an inferred reaction. Furthermore, because the second reaction produces the substrate for the first reaction, the two reactions are linked together in the 'Phosphatidylinositol phosphate metabolism' pathway.

We carefully reconciled the pathway organization during integration of the three reference models by comparing the reactions in each pathway. Thus, the pathway organization in MetaFishNet follows biochemical conventions wherever possible. Yet, over 600 reactions still do not map directly to these reference pathways. Since pathways can be viewed as modules within a metabolic network [33], we extracted network modules from these reactions using a modularity algorithm [34]. The resulting modules were manually inspected to either become a new pathway, to merge with an existing pathway, or to be invalidated. Meanwhile, individual reactions were attached to a pathway when they connect metabolites in that pathway. This combined procedure of module finding and manual curation was repeated iteratively until no further change could be made.

**Figure 2:**
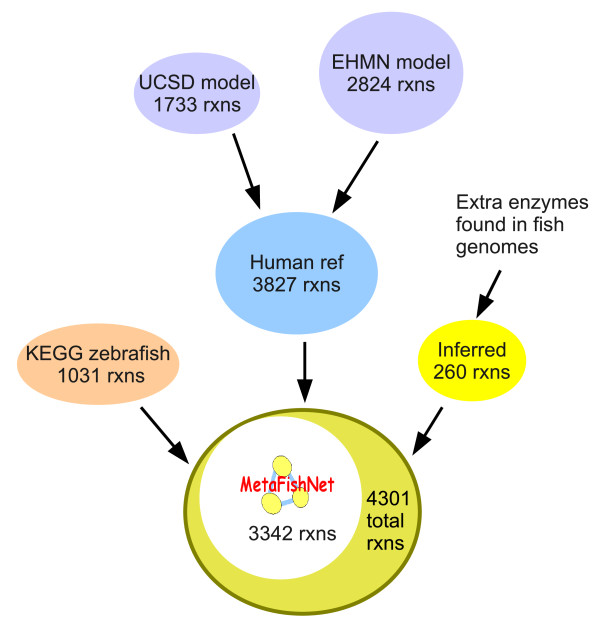
**Data integration at reaction level for MetaFishNet**. The UCSD and EHMN models were merged into a human reference network, which was then merged with the KEGG zebrafish model and newly inferred reactions based on genome sequences. The total reference model has 4,301 reactions, while 3,342 reactions are included in the fish metabolic network.

Even though this model contains data specific to each of the five fish species, we choose to present a combined fish metabolic network model because a) a combined model will be more useful for other under-represented fish species; b) genome annotations are far from perfect - combining five genome sequences will reduce the chance of missing true metabolic genes. For example in the TCA cycle, we did not find ATP citrate synthase in the zebrafish genome, nor succinate-CoA ligase in the *Tetraodon *genome (Ensembl 51). Since these are critical enzymes in a central pathway, these missing enzymes reflect annotation errors. The combined model is thus more comprehensive than using any single species alone (Additional file 2). In total, 911 enzymes, 3,342 reactions and 115 pathways are included in MetaFishNet version 1.9.6. Data integration at the reaction level is shown in Figure 2. All MetaFishNet pathways are given in Additional file 3, reaction data in Additional file 4 and SBML (Systems Biology Markup Language) distribution in Additional file 5.

A MySQL database was set up to host MetaFishNet data. As we elected to use Google App Engine to host the project website [35], a port to Google BigTable database is actually behind the website. The website supports browsing and queries of data at various levels, with graphic display of all pathways. Utility programs in MetaFishNet include 'SeaSpider' for sequence analysis, 'FishEye' for pathway visualization, and 'FisherExpress' for pathway enrichment analysis. SeaSpider is used for both the initial construction and for mapping new sequences to MetaFishNet. FishEye was developed because 1) KEGG graphs can no longer support the much expanded network, and 2) an automatic pathway visualization tool is of great general interest by itself. Our project website provides links to download these programs and model data.

### Metabolic genes show less evolutionary diversity

It is now widely accepted that teleost fish underwent an extra round of genome duplication after their evolutionary separation from the mammalian line [36,37]. Genome duplication is an important mechanism for generating gene diversity, as the extra copy can evolve more freely than the single copy before duplication. Only a small portion of these duplicated genes would gain new functionality and remain, while most duplicated genes got lost over time.

When comparing the fish metabolic genes in MetaFishNet to their human orthologs, we have noticed that the level of ortholog mapping differs between metabolic genes and other genes. As seen in Table 2, for the identifiable orthologs, most of the fish species have over 10% more genes than humans, yet the percentages of extra duplicated metabolic genes are significantly less. The final numbers may vary when the genomes are more accurately annotated. Still, these data suggest that metabolic genes are better conserved between human and fish than other genes. This suggests that a core metabolic network was established early in evolution: by the time of the genome duplication in fish, the central metabolic machinery was already well tuned and left little room for changes. By implication, research on some fish metabolic pathways may be easily extrapolated to human.

**Table 2:** Comparisons between fish and human orthologs

Species	Extra duplicated genes (%)	Extra duplicated metabolic genes (%)
Zebrafish	15.4	0.6
Medaka	8.9	1.5
Takifugu	12.2	3.8
Tetraodon	14.4	5.8
Stickleback	11.9	4.5

An extra round of genome duplication produced more genes in fish than human. The number of total human orthologs found in a fish species is typically around 12,000, as analyzed from Ensembl data.

### Comparison between human and fish metabolic pathways

**Figure 3:**
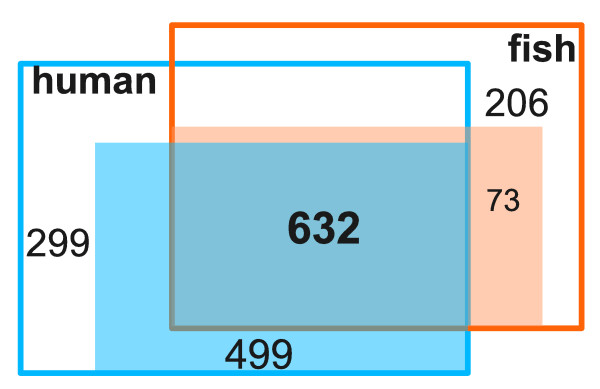
**Metabolic enzymes in common between human and fish**. Among the 1,430 human enzymes compfiled from ExPASy and BRENDA [91] databases, 1,131 are included in the human metabolic models (shaded in light blue). Among the 911 enzymes found in fish genomes, 705 are included in MetaFishNet reactions (shaded in salmon). In the models, 632 enzymes are shared between human and fish. The disparity of numbers reflects that human enzymes are better annotated than fish. Please note that isozymes are not distinguished here.

Multiple genes may have the same catalytic activity (isozymes), differing only in their sequences or regulatory contexts. We do not distinguish isozymes in this study, but leave them for future refinement. At the enzyme level, we have identified 911 enzymes from fish genomes. They overlap with the human data by 772 enzymes (Figure 3; Additional file 6 gives a complete list of these enzymes). The true overlap may be greater because the EC numbers in fish were computationally inferred, and are not as well curated as human ECs. We can nonetheless start making some comparisons between human and fish at the pathway level.

Over 50% of the enzymes are in common between human and fish for the majority of the pathways. Table 3 shows the most and least conserved pathways between humans and fish, in terms of the numbers of overlapping enzymes. Since most biomedical research in fish aims to extend the results to human, this pathway comparison reveals important information on how well fish may model human on a specific subject. For instance, fish may be a good model for studying vitamin B9, but probably a poor model for studying vitamin C.

**Table 3:** Comparisons between fish and human metabolic pathways

Most conserved pathways				
**Pathway**	**Human ECs**	**Fish ECs**	**Overlap**	**Ratio**

1- and 2-Methylnaphthalene degradation	2	3	2	1
Hyaluronan metabolism	3	3	3	1
Sialic acid metabolism	18	18	18	1
Hexose phosphorylation	5	5	5	1
Electron transport chain	4	5	4	1
Limonene and pinene degradation	3	4	3	1
Proteoglycan biosynthesis	16	16	16	1
Glycosphingolipid biosynthesis - ganglioseries	18	17	17	0.94
N-Glycan degradation	8	7	7	0.87
Di-unsaturated fatty acid beta-oxidation	7	6	6	0.85
Vitamin B1 (thiamin) metabolism	7	6	6	0.85
Glycosphingolipid metabolism	28	24	24	0.85
Glutamate metabolism	14	12	12	0.85
TCA cycle	18	15	15	0.83
Vitamin B9 (folate) metabolism	17	14	14	0.82
Linoleate metabolism	11	9	9	0.81

**Least conserved pathways**				

**Pathway**	**Human ECs**	**Fish ECs**	**Overlap**	**Ratio**

Phytanic acid peroxisomal oxidation	13	5	5	0.38
Glycosylphosphatidylinositol(GPI)-anchor biosynthesis	3	1	1	0.33
Vitamin H (biotin) metabolism	6	2	2	0.33
Vitamin B12 (cyanocobalamin) metabolism	3	2	1	0.33
Glyoxylate and Dicarboxylate metabolism	7	2	2	0.28
Pentose and Glucuronate interconversions	9	2	2	0.22
Ascorbate (vitamin C) and aldarate metabolism	8	1	1	0.12

The ratio is the number of shared ECs over the number of human ECs. Only pathways with three or more enzymes were considered. The complete comparison is given in Additional file 9. Please see Discussion section on the bias towards human data. The sizes of fish pathways may grow with improved annotation, but this is unlikely to change the ratios because all overlapping enzymes are already included here.

In the sizable pathway, 'proteoglycan biosynthesis', all 16 enzymes are common between human and fish. This suggests that the whole pathway may be identical between human and fish. Impairment of the proteoglycan biosynthesis pathway is responsible for a major class of enzyme deficiency diseases, mucopolysaccharidosis. Seven clinical types, including Hurler syndrome and Hunter syndrome, have been identified in this class, depending on defects of different enzymes in the pathway (Online Mendelian Inheritance in Man [38]). Given the great similarity between human and fish in this pathway, small fish, with their high throughput capacity, may be a good model for studying mucopolysaccharidosis.

**Figure 4:**
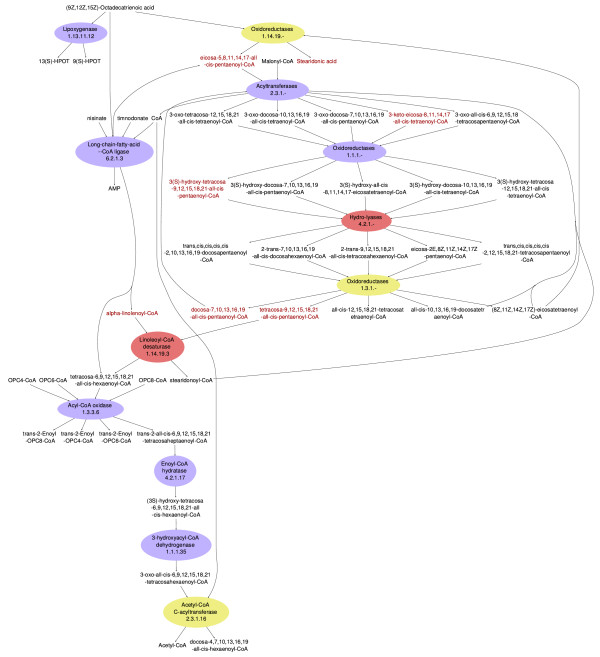
**Omega-3 fatty acid pathway**. The human omega-3 fatty acid metabolism pathway is composed of 12 enzymes. The enzymes colored in red are not found in fish. The three enzymes in yellow are in the gene families found in fish, but the presence of these specific enzymes is not clear. This shows that fish lack enzymes to convert the intermediate metabolites, which are the source of omega-3 fatty acids important to human health. The common omega-3 fatty acid variants are in red font.

Omega-3 fatty acids are deemed essential nutrients, boosting a popular dietary preference for fish and fish oil consumption. But fish, just like humans, do not produce omega-3 fatty acids *per se *- they accumulate them from their diet, algae [39]. However, the molecular mechanism of this omega-3 fatty acid accumulation is still unidentified. A theoretical explanation is now provided by our MetaFishNet model. As shown in Figure 4, compared to the human omega-3 fatty acid metabolism, fish lack enzymes such as linoleoyl-CoA desaturase in the pathway. As a result, fish can easily process the metabolites in the top and bottom parts of the pathway, but not the intermediate metabolites, which will then accumulate to a high level. In fact, these intermediate compounds include variants of most of the common omega-3 fatty acids, such as alpha-Linolenic acid, Stearidonic acid, Eicosatetraenoic acid, Eicosapentaenoic acid, Docosapentaenoic acid and Tetracosapentaenoic acid. It will be interesting to see if this computationally generated hypothesis will be supported by experimental data.

### Several metabolic pathways are misregulated in zebrafish liver cancer

We next demonstrate the application of MetaFishNet model to the analysis of gene expression data in a case of zebrafish as a cancer model. Gong and coworkers conducted microarray experiments to examine the similarity between zebrafish and human liver tumors at the level of gene expression [40]. Although they found the overlapping of gene expression was statistically significant, in-depth data analysis was limited to Gene Set Enrichment Analysis (GSEA) and to two signaling pathways (Wnt-beta-catenin and Ras-MAPK). We shall demonstrate here that MetaFishNet is a valuable addition to the arsenal of microarray data analysis.

The microarray data from [40] were retrieved from Gene Expression Omnibus (GEO [41]) via accession number [GEO:GSE3519]. The arrays contained 16,512 features, with 10 tumor samples and 10 control samples. Significance Analysis of Microarrays (SAM [42]) was used to select 1,888 differentially expressed clones between tumor samples and controls with a False Discovery Rate under 0.01. (These selected clones are comparable to the 2,315 clones selected by a less mainstream method in the original paper.) The pathway analysis component in MetaFishNet is FisherExpress, which maps the selected genes to enzymes and then to corresponding pathways via queries to the MetaFishNet database. Fisher's Exact Test is used to compute the significance of enrichment of metabolic pathways.

The result, shown in Table 4, suggests that several metabolic pathways are misregulated in zebrafish liver cancer. The identification of the glycolysis and gluconeogenesis pathway reflects the adaptation of tumor cells to aerobic glycolysis, known as the hallmark 'Warburg effect', which also alters pathways closely related to gluconeogenesis, such as butanoate metabolism [43,44]. The reprogramming of metabolism in tumor cells is also believed to generate toxic byproducts [43], in particular elevated levels of reactive oxygen species [45]. The downregulation of xenobiotics metabolism and ROS detoxification reflects these impaired cellular functions in tumor tissues. The involvement of tyrosine metabolism in tumor cells is not clear, but may possibly be related to their excessive tyrosine kinase activities [46,47]. Tryptophan metabolism is known to be part of the immune suppression mechanism by tumor cells [48]. The significance of leukotriene metabolism could come either from tumor cells that use leukotrienes in their strategies for survival, proliferation and migration, or from the inflammation of surrounding tissues [49].

**Table 4:** Metabolic pathways that are affected in zebrafish liver cancer with *P*-value < 0.05

MetaFishNet pathway	Selected enzymes	Enzymes in pathway	*P*-value
ROS detoxification	2	2	0.002
3-Chloroacrylic acid degradation	2	2	0.002
Tyrosine metabolism	8	55	0.002
Xenobiotics metabolism	3	8	0.004
Glycolysis and Gluconeogenesis	6	44	0.013
Fatty acid metabolism	3	13	0.019
Butanoate metabolism	3	14	0.023
Leukotriene metabolism	3	17	0.040
Tryptophan metabolism	4	29	0.040
Ascorbate (vitamin C) and aldarate metabolism	1	1	0.046
Glycosylphosphatidylinositol(GPI)-anchor biosynthesis	1	1	0.046

**Figure 5:**
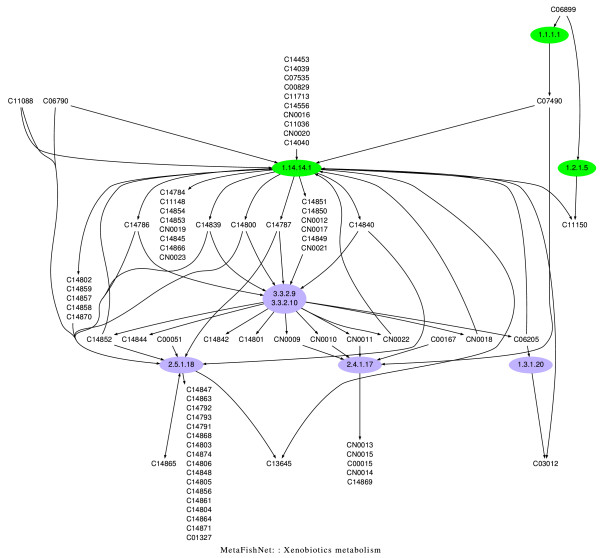
**The xenobiotic metabolism pathway in zebrafish liver cancer**. The three downregulated enzymes, colored in green, are 1.2.1.5, aldehyde dehydrogenase (AF254954); 1.1.1.1, alcohol dehydrogenase (AF295407); 1.14.14.1, cytochrome P450 (AF057713, AF248042). Fully annotated graphs for all pathways can be found on project website [35].

Fatty acid metabolism is also well known to be involved in cancer biology [43,50]. However, the selection of the fatty acid metabolism pathway in our analysis came from three enzymes it shares with the leukotriene metabolism pathway. Pathway overlap is an inherent limit of this type of analysis, that can only be clarified by further investigation. Several Glycosylphosphatidylinositol(GPI)-anchor proteins are already used as markers for liver cancer [51-53], making (GPI)-anchor biosynthesis an interesting pathway to investigate. The MetaFishNet model thus has been shown to be a valuable tool to identify significantly regulated pathways in expression data. In addition, the regulations can be visualized in the context of each pathway, as exemplified in Figure 5, to facilitate mechanistic studies.

### Comparison to KegArray and KEGG pathways

KEGG also offers an expression analysis tool, KegArray [21], which may be used to map differentially expressed genes to zebrafish pathways. For example, the 1,888 selected clones in zebrafish liver cancer in Section 2.4 can be converted to UniGene identifiers and input to KegArray (version 1.2.3). The result is a list of 49 metabolic pathways that match from one to five differentially expressed enzymes (Additional file 7). This is a rather long list, containing about half of all pathways, which raises the question of false positive rate. The problem is caused by the fact that KegArray does not include any pathway statistical analysis, which is important for ranking the significances and reducing false positives at the individual gene level. Pathway enrichment analysis usually takes one of two forms: 1) feature selection followed by set enrichment statistics, such as presented in this paper and 2) competitive statistics without prior feature selection. The best known example of the latter is GSEA [22], which uses Kolmogorov-Smirnov statistics to rank pathways according the positional distribution of member genes. As the MetaFishNet model itself is not tied to any statistical method, we also offer a gene matrix file to be used with GSEA, downloadable at our project website.

Ultimately, the quality of pathway data determines the quality of analysis. MetaFishNet, with 3,342 reactions over the 1,031 reactions in KEGG zebrafish model, not only allows applications to other fish species, but also improve the data for zebrafish. A better comparison between the KEGG zebrafish model and MetaFishNet is to use the same enrichment statistics. That is, we use the KEGG pathways in our software instead of MetaFishNet pathways to reanalyze the zebrafish liver cancer data in Section 2.4. The result is shown in Additional file 8. In comparison to Table 4, leukotriene metabolism and ROS detoxification pathways are missing in the KEGG result as they are absent in the KEGG model.

Xenobiotics metabolism is a pathway that is improved from five enzymes in KEGG to eight enzymes in MetaFishNet. Accordingly, the MetaFishNet pathway has three hits while the KEGG pathway has two hits. The Methane metabolism pathway, nonexistent in MetaFishNet, was also identified in KEGG. The KEGG Methane metabolism pathway is rather a bacterial pathway that is mapped to zebrafish with only three reactions. Reaction R06983 is catalyzed by an enzyme (1.1.1.284) that is yet to be confirmed in any fish genome. Reaction R00945 converts 5,10-Methylenetetrahydrofolate to Tetrahydrofolate, thus is assigned to vitamin B9 (folate) metabolism pathway in MetaFishNet. This leaves only one reaction, which does not justify a pathway in MetaFishNet. We think the improved data and pathways in MetaFishNet will benefit downstream studies.

### MetaFishNet analysis of cadmium exposure in sheepshead minnows

Finally, we apply MetaFishNet to a fish species with little functional data. Sheepshead minnow (*C. variegatus*) is a common, small estuarine fish that is found along the Atlantic and Gulf coasts of the United States. The US Environmental Protection Agency has adopted *C*. *variegatus *as a model organism for studying pollution levels in estuarine waters [54]. We have designed a custom DNA microarray with 4,101 clones for sheepshead minnows. Sheepshead minnow larvae were exposed to cadmium, a heavy metal pollutant, for seven days in a controlled laboratory experiment. DNA microarrays were used to measure their RNA expression. Even though each biological replicate was a pool of 80 individuals, only three biological replicates per group were included in this microarray experiment. The analytical power at the gene level was also weakened because the samples were extracted from whole bodies instead of specific tissues. Indeed, with FDR < 0.05 in SAM, only four clones were selected as significant, including metallothionein, which has been extensively reported to be upregulated by cadmium exposure [55,56].

Another problem is the poor annotation of these microarrays. Less than 40% of our sheepshead minnow clones carry sequence homology to known genes, a situation typical for many fish species that limits the functional information from gene expression.

**Figure 6:**
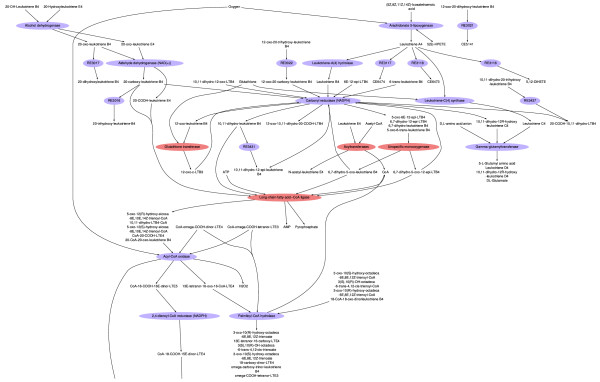
**The leukotriene metabolism pathway as modulated by cadmium exposure in sheepshead minnow**. Four upregulated enzymes are colored in red. Only a partial pathway is shown. Some metabolites are connected by reaction IDs when the enzymes are not known.

To analyze the data in MetaFishNet, we first selected 325 differentially expressed clones between the treated group and control group by Wilcoxon's rank sum test (*P *< 0.05). This is a less stringent selection, but additional statistical strength is gained at the pathway level by incorporating collective pathway information. Sheepshead minnow clones were then mapped to MetaFishNet by sequence comparison via SeaSpider. MetaFishNet pathway enrichment was computed again by Fisher's Exact Test and the result is shown in Table 5. The pathways in Table 5 again have overlaps, among which are CYP1A and glutathione S-transferase (GST). The induction of CYP1A and GST by cadmium is in concordance with previous reports [57-61]. Both CYP1A and GST are pivotal detoxification enzymes, and central players in xenobiotics metabolism. The fact that these genes are picked up by pathway analysis and not by SAM demonstrates the improved strength of pathway analysis. The upregulation of four enzymes, CYP1A, GST, acyltransferase and long-chain-fatty-acid-CoA ligase, is indicative of the activation of leukotriene metabolism pathway by the commonly observed inflammation induced by cadmium exposure (Figure 6).

**Table 5:** Metabolic pathways that are affected by cadmium exposure in sheepshead minnows with *P*-value < 0.05

MetaFishNet pathway	Selected enzymes	Enzymes in pathway	*P*-value
Leukotriene metabolism	4	17	0.001
Fatty acid metabolism	3	13	0.005
Omega-3 fatty acid metabolism	2	7	0.016
Squalene and cholesterol biosynthesis	3	20	0.018
Xenobiotics metabolism	2	8	0.021
Omega-6 fatty acid metabolism	2	10	0.032
Tryptophan metabolism	3	29	0.049

In conclusion, MetaFishNet adds extra functional insight into the otherwise very limited data analysis available for non-model species.

## Discussion

We have presented the first genome-wide fish metabolic network model. The first and primary role of our MetaFishNet model is a bioinformatic tool for analyzing high throughput expression data. Two case applications of pathway enrichment analysis are included in this report. Pathway analysis offers two advantages: it is less susceptible to noise than analysis at the level of individual genes, and gives contextual insights to biological mechanisms [62,63]. MetaFishNet has demonstrated good promise to bring these advantages into fish studies. By combining data from five fish genomes, our model overcomes some of the coverage problems in individual genome annotations. However, this also masks the difference between these fish species. While this combined model is recommended for gene expression analysis, species specific data should be consulted for more specific genetic and biochemical studies (available at the project website).

A new visualization tool (FishEye) was developed in this project to draw pathway maps automatically.

Even though visualization tools are abundant, there is a particular challenge to balance automation with the kind of clarity desired in a metabolic map. KEGG, and many other pathway databases, creates graphs manually. Hence, all downstream automatic programs in fact depends on the original manual versions.

CellDesigner [64] is an excellent tool, but essentially is for manual editing. On the other hand, CytoScape [65] and VisANT [66] can do automatic drawing, but their results tend to be cluttered and difficult for detailed studies of metabolic pathways. FishEye is a light-weight and flexible Python program based on the widely used Graphviz package from AT&T Research Labs [67]. Rgraphviz [68] is a similar package that offers R binding of Graphviz. The unique strength of FishEye is its optimization for rendering biological pathways via analyzing network structure and labels. FishEye has worked successfully for this project. Its limit seems to be only challenged by two pathways that exceed 400 edges. For these cases, a 'zoom' feature was introduced to reduce the cluttering of edges. We hope that FishEye will find uses in other similar contexts.

We should emphasize that the knowledge of vertebrate metabolism is still very incomplete. This is already evident when considering the obvious differences between the two human models [20,31]. With the assistance of modularity analysis, we constructed several new pathways that were not present in the reference models. For instance, our analysis showed that all 18 enzymes in a newly identified 'sialic acid metabolism' pathway are in fact present in both fish and humans. This shows both the strength of our construction approach and the incompleteness of current models. In general, when one compares the fish pathways versus human pathways (Table 3), the latter seem to contain more enzymes. Because the UCSD and EHMN projects were intensively curated and contained many more data than previous models, a combined human dataset in this project is unlikely to be surpassed by any computational model. Due to the bias in annotations, fish enzymes that have human homologs are also more likely to be incorporated into MetaFishNet. On the other hand, as discussed above, we actually further augmented the human data through constructing MetaFishNet (demonstrated in Additional file 9).

As a first generation model, MetaFishNet will need much refinement to fully realize the power of a genome-wide metabolic model. Traditionally, metabolism was studied piecemeal by dissecting enzyme activities and tracking metabolites. Powerful new tools have now been introduced to genome-wide models [69,70]. For example, mass balance of metabolites can be achieved by a combination of the stoichiometrics of reactions and physiologically plausible kinetics and thermodynamics of pertinent enzymatic reactions. Even with incomplete information, system constraints such as metabolite flux can be deduced. Missing reactions in the model can be inferred in a similar fashion. While improvements can be expected from accumulating data and annotations, with this MetaFishNet framework now in place, it is possible to design systematic experiments to define and refine fish metabolome. That is, metabolic constraints can be inferred from MetaFishNet model; experimental data can then be gathered, utilizing mutants or knockouts, to verify and update the model iteratively [71-73]. Such works will lead the way for species specific models.

Recent studies have shown that gene expression data, combined with metabolic network models, can successfully predict metabolic flux regulation in specific biological contexts [74-76]. This opens up an exciting opportunity to advance fish metabolic modeling. Finally, metabolic networks are a natural platform to integrate multiple high throughput data types. For example, Yizhak *et al*. used a *E. coli *metabolic network [30] to combine proteomic data with metabolomics to predict knockout phenotypes [77].

Connor *et al*. combined transcriptomics and metabolomics on Ingenuity's human metabolic pathways http://www.ingenuity.com to identify type two diabetes markers [78]. With the advancing of fish omics, in particular metabolomics [79-81], MetaFishNet is in a good position to fulfill a similar important role for fish studies. The rate of discovery can be greatly accelerated when MetaFishNet is combined with these high throughput technologies.

## Methods

### Identification of fish metabolic enzymes and sequence analysis

All cDNA sequences of the five fish species were retrieved from the Ensembl database [82]. Identification of metabolic genes was accomplished by Gene Ontology (GO) computation [83]. Among the five fish species, only zebrafish had good GO annotations. Sequences from the other four species were analyzed by SeaSpider, our sequence analysis tool. The queries to SeaSpider are first directed against zebrafish sequences, then against reference sequences in the GO database. When homology is found (BLAST E-value under 1E-5 and a minimum 33 of identical bases in local alignment), GO terms are assigned to the sequence in query. All genes with a GO term under the tree of metabolism are considered to be metabolic genes. Even though this initial selection is overly inclusive - for example, transport proteins can also get a GO term under metabolism - only genes that can match to EC numbers are used in MetaFishNet construction. We inferred EC numbers in two ways. The first approach was to carry over EC numbers from human orthologs. The orthologous relationships between fish and human genes were adopted from Ensembl, which has thoroughly computed ortholog/paralog relationships based on the phylogenetic tree of the gene family. Human EC to gene associations were parsed from the ExPASy database [84] and the EHMN data [31]. The second approach of EC inference was through annotations in the GO database by similarity to the enzyme consensus sequences, which have been constructed across species. It should be pointed out that the EC numbers in MetaFishNet are tentative - the Nomenclature Committee of IUBMB actually requires strict experimental evidence for assigning an official EC number.

### Integration of reference reaction data

We first integrated the two high-quality human metabolic models [20,31]. The zebrafish metabolic model was then extracted from KEGG, and combined into the reference data. The UCSD model contained 1,496 genes and 3,311 reactions, counting transport reactions and compartmentalization. A highlight of this work was the manual curation of literature supports, which was labor intensive but improved the data quality.

The EHMN model has 2,322 genes and 2,824 reactions (excluding transport reactions). The EHMN model included previous metabolic data from all major databases, and streamlined the identities of compounds. Automatic extraction of metabolic models from KEGG has been a challenge. Even though KEGG offers an XML (Extensible Markup Language) distribution (called KGML) of its pathways, molecular interactions were mixed with visual elements in these KGML les. KEGG API (Application Programming Interface) was also limited by not distinguishing reactants from products. We developed a practical solution by combining KGML files and KEGG API, where KGML defines the scope of reactions and API confirms relationships. Our Python script, leveraging on SBML libraries, successfully parsed out the 101 zebrafish metabolic pathways from KEGG (retrieved March 24, 2008), with 517 ECs and 1,031 reactions.

The integration of three models was at both the reaction and pathway levels. Two reactions were considered identical when they have the same enzymes and major compounds. To gain the most compatibility, EC numbers and KEGG compound IDs were used wherever possible. The conventional pathways in MetaFishNet primarily followed the pathway organization in EHMN. Pathways were merged if they shared a significant number of common reactions. Different naming styles were reconciled. For example, the 'Cholesterol Metabolism' pathway in the UCSD model overlaps with the 'Squalene and cholesterol biosynthesis' pathway in the EHMN model by 14 enzymes and 16 reactions. The two pathways were merged during the integration of the two human models. All three reactions in the KEGG zebrafish pathway 'Terpenoid biosynthesis' are included in the human 'Squalene and cholesterol biosynthesis' pathway and were therefore merged with the latter. Nine out of 11 enzymes in the zebrafish 'Biosynthesis of steroids' pathway are included in the human Squalene and cholesterol biosynthesis pathway, and were therefore merged as well. Complete lists of pathway reorganization are given in the Additional file 1. The current model does not take into account cellular compartmentalization.

### Ab initio construction, modularity analysis and manual curation

Among the 911fish enzymes identified in this project, 561 could be matched to the reference data. For the remaining 350 enzymes, their associated compounds were retrieved from the KEGG LIGAND database wherever available. These enzyme-compounds interactions formed 260 newly inferred reactions. Since there was no way to distinguish reactants from products in these inferred metabolic data, the directions of these reactions were treated as unknown. These newly inferred reactions, plus the isolated reactions from the reference data, were subjected to a combined approach of module-finding and manual curation. We adopted an algorithm by Mark Newman, which partitions network modules according to the eigenvectors of a characteristic matrix for the network [34]. The modularity program produced a number of candidate modules, which were then manually inspected for pathway organization. This process iterated until no further change could be made. Isolated reactions were also inspected to determine if they could be attached to existing pathways. At this stage, a number of redundant reactions from UCSD were removed from the model, and pathways with too few reactions were dismantled to isolated reactions. Through this approach, the 'sialic acid metabolism', 'dynorphin metabolism', 'electron transport chain', 'parathion degradation' and 'hexose phosphorylation' pathways were created from *ab initio *construction, while a number of modules were organized into existing pathways (Additional file 1).

### Pathway visualization

FishEye, our pathway visualization tool, is built on Networkx and PyGraphviz [85]. It extended a development version of Networkx to support bipartite networks. Many details of styling are manipulated through mid-level markups. In order to keep pathway graphs less cluttered, we did a number of optimizations. Two versions of pathway graphs are offered, one with EC numbers and compound IDs (for example Figure 5) and one with enzyme names and compound names (for example Figure 4 and 6). Both versions for all pathways are available at the project website. Similar edges in a pathway can be merged in the visualized graph, and long names are wrapped. A common practice in the field is to omit all currency metabolites, as they bring on an excessive number of edges. We adopted the list of currency metabolites in [86], as it conforms identically to the most connected nodes in MetaFishNet. However, we leave the inclusion of currency metabolites optional, depending on their degrees in specific pathways.

### Expression profiling of sheepshead minnows exposed to cadmium

We have previously generated Suppressive Subtractive Hybridization libraries for sheepshead minnows, and sequenced over 10,000 clones [87]. Based on these sequences, we designed a DNA microarray of 14,494 probes for 4,101 clones. All probes were synthesized on microarray chips by Nimblegen Inc. with four replicates.

Exposures and animal sampling were performed as previously described [88,89]. Cadmium (0.3 mg/L) was administered to sheepshead minnow larvae at 24 hours post hatch via precision syringe pumps in an intermittent flow-through system [90]. The study included three biological replicates, each containing 80 larvae in four cups. After seven days of exposures, whole larvae were sacrificed and stored in RNAlater (Ambion Inc., Austin, TX). Total RNAs were then extracted using the phenol/chloroform method, and treated with DNase. The purified RNAs were checked by NanoDrop and BioAnalyzer for quality assurance. The labeling of RNAs was carried out according to recommendation by Nimblegen Inc. In short, mRNAs were converted to double-strand cDNA. Cy3-labeled random nonamers were used as primers for DNA polymerase reaction, which produced labeled DNA targets off the double-strand cDNA. These labeled targets were purified and hybridized to microarrays. The resulted fluorescent intensities were corrected by quantile normalization. Data at the probe level were averaged over on-slide replicates, with outliers removed. The expression values at the gene level were summarized as the geometric mean of its probe intensities.

## Abbreviations

API: application programming interface; EC: enzyme commission; EHMN: Edinburgh human metabolic network; FDR: false discovery rate; GEO: gene expression omnibus; GO: gene ontology; GSEA: gene set enrichment analysis; IUBMB: international union of biochemistry and molecular biology; KEGG: Kyoto encyclopedia of genes and genomes; KGML: KEGG markup language; SAM: significance analysis of microarrays; SBML: systems biology markup language; UCSD: University of California at San Diego; XML: extensible markup language.

## Authors' contributions

SL designed and performed most of the computational work. MB designed and supervised the experimental study. AP and MB provided critical guidance of the project and valuable discussions. CSM performed the cadmium exposure of sheepshead minnows. NBP and RR dissected the fish, extracted and labeled RNA. AP coordinated the sheepshead minnow microarray design and experiments. SL and MB wrote the manuscript.

## Supplementary Material

Additional file 1 **Supplemental method **[92-101]. Click here for file

Additional file 2 **Species specific statistics of pathways**. Click here for file

Additional file 3 **List of MetaFishNet pathways**. Click here for file

Additional file 4 **MetaFishNet reaction data**. Click here for file

Additional file 5 **SBML distribution of MetaFishNet pathways**. Click here for file

Additional file 6 **Fish and human enzymes**. Click here for file

Additional file 7 **Analysis of zebrafish liver cancer data by KegArray**. Click here for file

Additional file 8 **Analysis of zebrafish liver cancer data by KEGG pathways and Fisher's exact test**. Click here for file

Additional file 9 **Complete comparison between fish and human metabolic pathways**. Click here for file
