# Supplementary material for: Constructing a fish metabolic network model
Source: Genome Biol. 2010 Nov 29;11(11):R115. doi: 10.1186/gb-2010-11-11-r115 (PMC3156954; doi:10.1186/gb-2010-11-11-r115)
Supplement: Additional file 1 — Supplemental method [92-101]. [file gb-2010-11-11-r115-S1.pdf]

Supplemental Method for

Constructing a fish metabolic network model

Shuzhao Li, Alexander Pozhitkov, Rachel A. Ryan, Charles S. Manning, Nancy Brown-Peterson and Marius Brouwer

1 Concepts of metabolic networks

In metabolic networks, there are two common types of nodes, enzymes and compounds; edges represent their interactions. Thus, metabolic networks are usually modeled as a class of "bipartite network", where an enzyme (or enzyme complex) is connected to another enzyme only via a common compound. Depending on the context of individual reactions, this compound may be the product of one reaction and the reactant of another reaction. The connecting edges also bear directionality from such reactions. An edge can go both ways if the reaction is reversible (Figure 1A, D).

In computer programming, a network can be represented either as an adjacency matrix or an adjacency list (Figure 1B, C). Both representations were used in the programs for MetaFishNet, depending on the situation. The number of edges attached to a node is called the "degree" of the node. Distribution of the degrees is often used to study the internal structure of networks. For example, the degrees in a random network follows the Poisson distribution. When the degree distribution in a network follows a power law, this network is a "scale-free" network [92]. Most biological networks, including metabolic networks, have been shown to be scale-free [33,93,94].

Modularity is a measure for community structures in a network. An influential mathematical definition of modularity was given by Newman and Girvan [95]. Ma et al. demonstrated that metabolic pathways can be detected by finding modularity in the larger networks [33]. Pathway organization was an important issue during the construction of the MetaFishNet model. We have implemented a modularity finding algorithm by Newman [34] to aid the pathway curation process.

A small number of molecules, for instance, water, oxygen and ATP, are present ubiquitously in metabolic reactions. They are called currency metabolites. They are usually removed from network analysis and visualization, which would be otherwise cluttered. What should be included in this list of currency metabolites is a matter of minor debate [26,96-98], and somewhat dependent on the exact pathway context. They usually correspond well to the metabolites of highest connection degrees. This is also the case in MetaFishNet (Table 1). The list by Holme and Huss [85], which is very close to that of Ma and Zeng [26], fits slightly better to our data and was adopted in this study. In previous metabolic models, currency

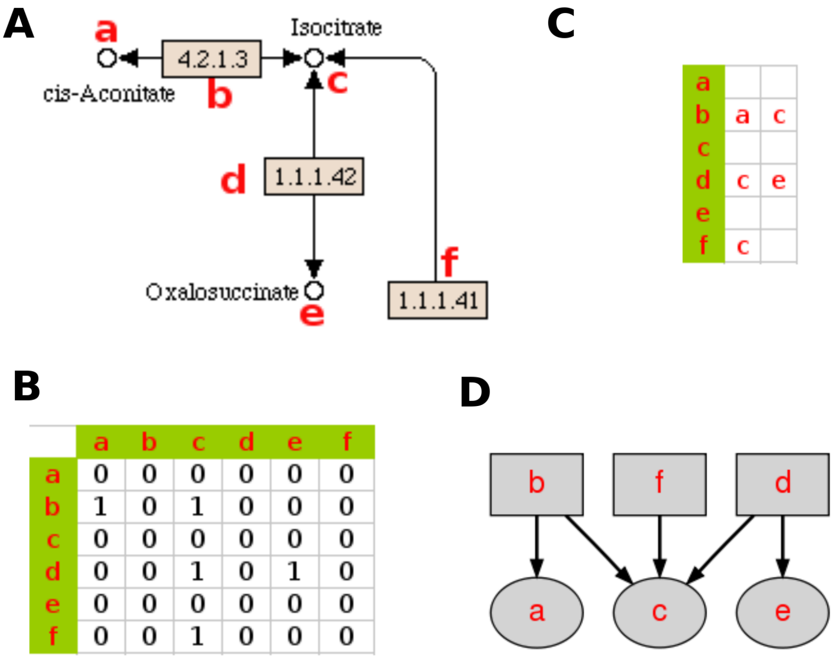

Figure 1: Representations of a network. (A) part of TCA cycle, as an example of metabolic network. (B) Adjacency matrix (C) Adjacency list (D) A different rendering of the same graph.

metabolites may or may not be included in a reaction description. Thus, we excluded currency metabolites from reaction comparisons and network modularity analysis.

2 SeaSpider, the sequence analysis tool

Sequence analysis plays several key roles in the MetaFishNet project. During the construction process, the genes from five fish genomes were analyzed for Gene Ontologies (GO), then the metabolic genes were identified by their GO categories. The identification of enzymes from fish genes, if without known human homologs, depends on the sequence similarity to consensus enzyme sequences. For the applications of MetaFishNet, sequence comparison is often the only way to identify the genes submitted by users. As illustrated in Figure 2, the *ab initio* annotation by *SeaSpider* associates genes to their GO terms wherever possible; while the other function maps users' genes onto MetaFishNet model. Different databases are used for these two functions. For *ab initio* annotations, new sequences are searched against the zebrafish sequence database first, then the generic GO sequence database. For sequences that do not have matches

Table 1: The hub (most connected) compounds in human metabolic network correspond to the currency metabolites designated in previous studies [26,85]. Compounds of highest degrees in the merged EHMN and UCSD models are listed in the left column. We thus adopted the list from Holme & Huss as currency metabolites for this study.

hub compounds in new data	Holme & Huss	Ma & Zeng
ATP	ATP	ATP
ADP	ADP	ADP
$NADPH$	$NADPH$	$NADPH$
$NADP^+$	$NADP^+$	$NADP^+$
NAD^+	NAD^+	NAD^+
$NADH$	$NADH$	$NADH$
P_i	P_i	P_i
PP_i	PP_i	PP_i
CO_2	CO_2	CO_2
H_2O	H_2O	H_2O
O_2	O_2	O_2
H^+	H^+	
NH_3		NH_3
		SO_4
H_2O_2		
CoA		
$Acetyl\text{-}CoA$		
UDP		
AMP		

in these local databases, *SeaSpider* queries them further to NCBI remotely. The last step does not introduce GO information, but makes *SeaSpider* a competent standalone application for annotating new gene sequences. For the mapping to MetaFishNet, new sequences are searched against all the metabolic genes used in MetaFishNet, then taken to the next step of pathway analysis.

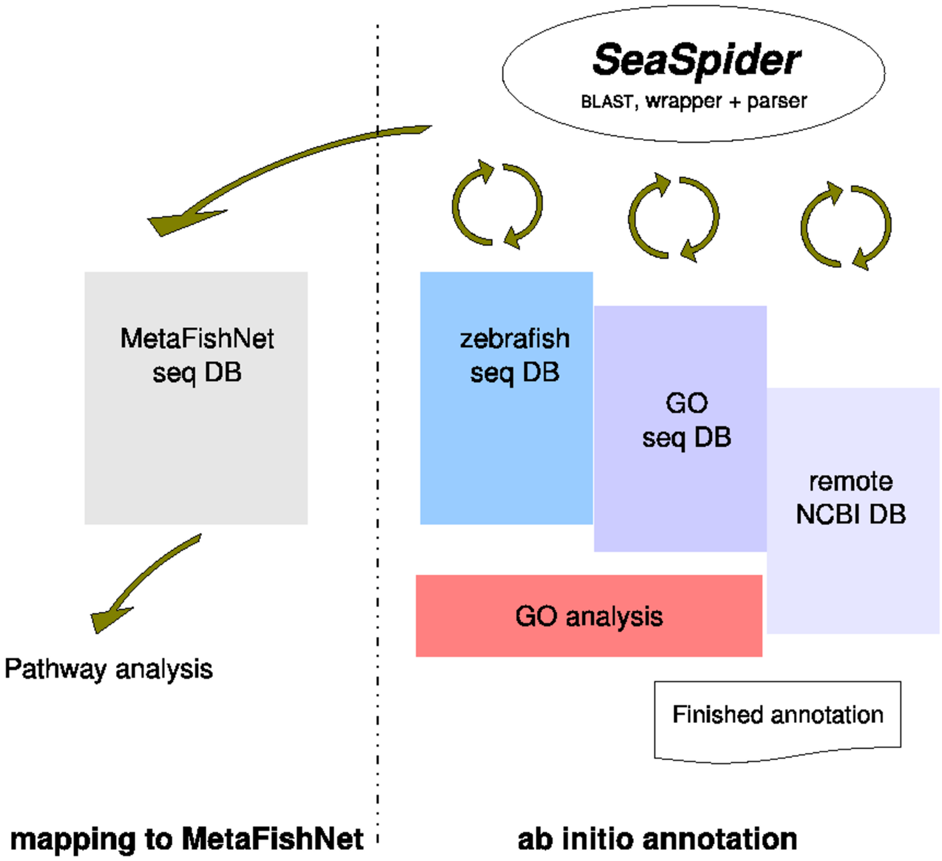

Figure 2: *SeaSpider* is used for both ab initio annotation and the mapping to MetaFishNet.

Gene Ontology

The whole set of Gene Ontology is modeled as a directed acyclic graph. When a GO term is assigned to a gene, the gene is automatically associated with all its upstream terms. They can come from all of the three major categories: biological process, molecular function and cellular component. It is common that a single gene is associated with dozens of GO terms. The relationships among these GO terms have to be tracked through the database provided by the GO Consortium. Since intense database queries are involved and the size of the complete GO database is manageable (about 400 MB), we keep and use a local copy of GO

database.

Zebrafish has good GO annotations, which came mostly from the ZFIN (ZebraFish Information Network [100,101]) project. The gene sequences from genomes of medaka, Takifugu, Tetraodon and stickleback were annotated by *SeaSpider*. A gene is considered "metabolic" when it is associated with the GO term "metabolic process" and a next step will be taken to find its appropriate Enzyme Commission (EC) number.

the SeaSpider program

The sequence search program BLAST [99] from NCBI is used as a component. SeaSpider wraps the input and output of BLAST, and parses the XML results. XML parsing is done using *ElementTree*, which in this case is more memory efficient than standard DOM (Document Object Model) tools. Python library *subprocess* is used to wrap BLAST. A newer version of this library should be used to avoid a problem in pipe buffering. Database queries are performed via Python binding to MySQL.

SeaSpider needs to record the status of its queries internally. This is achieved via Python *shelve*, which is a serialized object database. The most memory consuming part of SeaSpider is the parsing of BLAST results in large batches. E.g., a batch query of 500 sequences may use over 500 MB. This is not a real concern on modern computers, and the batch size can be decreased to accommodate less powerful hardware.

SeaSpider is organized as a Python package. It can be run directly from a command line Shell, or imported into other Python applications. We have used SeaSpider to annotate sequences from *Cyprinodon variegatus* and *Litopenaeus vannamei*. The full version of SeaSpider needs supports from several databases. A trimmed version that does not require database support, seaspider-lite, is provided to perform the mapping of user sequences to MetaFishNet genes.

3 Data integration and Pathway reconsolidation

The key of integrating different data sources is a unified representation of reactions, because once all reactions are in place, the new network can be recovered by connecting the reactions. In practical terms, the unified representation means all enzymes are coded in EC numbers and all compounds are in KEGG compatible IDs (KEGG has one of the largest collections of compounds). The nomenclature of compounds is rarely consistent across literature. The EHMN project did a good job to reconcile them with KEGG IDs. For the compounds not found in KEGG, the EHMN project assigned new IDs consistent with KEGG style. Reactions from the two human models were extracted by a combination of parsing XML files (SBML) and

```
<reaction id="R00549" reversible="false">
<listOfReactants>
<speciesReference species="C00002" stoichiometry="1" />
<speciesReference species="C00255" stoichiometry="1" />
</listOfReactants>
<listOfProducts>
<speciesReference species="C00008" stoichiometry="1" />
<speciesReference species="C00061" stoichiometry="1" />
</listOfProducts>
</reaction>

<reaction id="R_RBFK" name="riboflavin kinase" reversible="false">
<listOfReactants>
<speciesReference species="M_atp_e" stoichiometry="1"/>
<speciesReference species="M_ribflv_e" stoichiometry="1"/>
</listOfReactants>
<listOfProducts>
<speciesReference species="M_adp_e" stoichiometry="1"/>
<speciesReference species="M_fmn_e" stoichiometry="1"/>
<speciesReference species="M_h_e" stoichiometry="1"/>
</listOfProducts>
</reaction>
```

Figure 3: SBML descriptions of the same reaction from EHMN (top) and UCSD (bottom) models. SBML defines reactants and products clearly. But the identifiers still need a unified nomenclature. Note that the UCSD example contains an extra proton, which is considered a currency metabolite and not included in the EHMN example.

flat files (corresponding to their original databases). SBML distributions usually have a complete definition of network relationships. But the definitions of enzymes and compounds have to be verified from their databases or flat files. Flat files of the UCSD model were obtained from personal communication (Jan Schellenberger at Palsson lab). XML parsing was done with xml.dom.minidom implementation in Python libraries. Identifiers were extracted from flat files with the help of regular expression. Typically, four steps were involved in merging models:

1. Unifying all identifiers to compatible formats, e.g., all enzymes to EC numbers and all compounds to KEGG compatible IDs.

2. Comparing pathways. Pathways were manually inspected to decide whether to merge or change if they meet any of the criteria: a) sharing more than 4 enzymes; b) sharing more than 60% of enzymes; c) having the same theme.

3. Comparing reactions, removing repetitive reactions. Two reactions were considered identical when they have identical enzymes and identical compounds excluding currency metabolites, because

currency metabolites might or might not be included in the original descriptions.

4. Manual inspection of merged data. E.g., some pathways are functionally identical but differ significantly in source models. Such pathways require manual merging.

Integration of two high quality human models

Table 2 shows the 49 pathways from the UCSD model (91 total) to be merged into the corresponding pathways in the EHMN model. Transport reactions from the UCSD model were excluded. The "Nucleotides" pathway in UCSD model was dismantled because it is covered by the "Purine metabolism" and "Pyrimidine metabolism" pathways in the EHMN model. In the merged result, pathway "CYP Metabolism" was merged into "Xenobiotics metabolism"; "Ascorbate and Aldarate Metabolism" and "Vitamin C metabolism" were merged to "Ascorbate (Vitamin C) and Aldarate Metabolism". The EHMN pathway "Urea cycle and metabolism of arginine, proline, glutamate, aspartate and asparagine" was too large so that the several overlapping smaller pathways in the UCSD model were adopted instead. The rest of pathways were not affected at this stage. In total, 2824 reactions from EHMN and 1859 reactions from UCSD were merged to 3953 reactions and 106 pathways.

Table 2: The pathways in UCSD model to be merged with corresponding EHMN pathways.

**EHMN pathway**	**UCSD pathway**
Aminosugars metabolism	Aminosugar Metabolism
Arachidonic acid metabolism	Eicosanoid Metabolism
Bile acid biosynthesis	Bile Acid Biosynthesis
Biopterin metabolism	Tetrahydrobiopterin
Butanoate metabolism	Butanoate Metabolism
C21-steroid hormone biosynthesis and metabolism	Steroid Metabolism
De novo fatty acid biosynthesis	Fatty acid elongation
Fructose and mannose metabolism	Fructose and Mannose Metabolism
Galactose metabolism	Galactose metabolism
Glycerophospholipid metabolism	Glycerophospholipid Metabolism
Glycerophospholipid metabolism	Triacylglycerol Synthesis
Glycine, serine, alanine and threonine metabolism	D-alanine metabolism
Glycine, serine, alanine and threonine metabolism	Glycine, Serine, and Threonine Metabolism
Glycolysis and Gluconeogenesis	Glycolysis/Gluconeogenesis
Glycosphingolipid metabolism	Sphingolipid Metabolism
Histidine metabolism	Histidine Metabolism
Lysine metabolism	Lysine Metabolism

Continued on next page

EHMN pathway	UCSD pathway
Methionine and cysteine metabolism	Cysteine Metabolism
Methionine and cysteine metabolism	Methionine Metabolism
Methionine and cysteine metabolism	Taurine and hypotaurine metabolism
N-Glycan biosynthesis	N-Glycan Biosynthesis
O-Glycan biosynthesis	O-Glycan Biosynthesis
Pentose phosphate pathway	Pentose Phosphate Pathway
Phosphatidylinositol phosphate metabolism	Glycosylphosphatidylinositol (GPI)-anchor biosynthesis
Phosphatidylinositol phosphate metabolism	Inositol Phosphate Metabolism
Porphyrin metabolism	Heme Biosynthesis
Porphyrin metabolism	Heme Degradation
Proteoglycan biosynthesis	Chondroitin / heparan sulfate biosynthesis
Purine metabolism	IMP Biosynthesis
Purine metabolism	Purine Catabolism
Purine metabolism	Salvage Pathway
Pyrimidine metabolism	Pyrimidine Biosynthesis
Pyrimidine metabolism	Pyrimidine Catabolism
Selenoamino acid metabolism	Selenoamino acid metabolism
Squalene and cholesterol biosynthesis	Cholesterol Metabolism
TCA cycle	Citric Acid Cycle
Tryptophan metabolism	Tryptophan metabolism
Tyrosine metabolism	Phenylalanine metabolism
Tyrosine metabolism	Tyrosine metabolism
Tyrosine metabolism	Tyr, Phe, Trp Biosynthesis
Valine, leucine and isoleucine degradation	Valine, Leucine, and Isoleucine Metabolism
Vitamin A (retinol) metabolism	Vitamin A Metabolism
Vitamin B1 (thiamin) metabolism	Thiamine Metabolism
Vitamin B2 (riboflavin) metabolism	Riboflavin Metabolism
Vitamin B3 (nicotinate and nicotinamide) metabolism	NAD Metabolism
Vitamin B5 - CoA biosynthesis from pantothenate	CoA Biosynthesis
Vitamin B6 (pyridoxine) metabolism	Vitamin B6 Metabolism
Vitamin B9 (folate) metabolism	Folate Metabolism
Vitamin H (biotin) metabolism	Biotin Metabolism

Merging KEGG zebrafish data

The merging of the KEGG zebrafish model with the human model followed the same procedure as above. Reactions are marked by fish or/and human according the presence of their enzymes in those species. The spontaneous reactions (without an enzyme) may be necessary for mass flow in metabolic pathways and were kept in MetaFishNet.

Table 3: The pathways in KEGG zebrafish model to be merged with corresponding human reference pathways.

**zebrafish pathway**	**human pathway**
Alanine and Aspartate Metabolism	Alanine and aspartate metabolism
Prostaglandin formation from arachidonate	Arachidonic acid metabolism
Omega-3 fatty acid metabolism	alpha-Linolenic acid metabolism
Aminosugars metabolism	Aminosugars metabolism
Androgen and estrogen biosynthesis and metabolism	Androgen and estrogen metabolism
Arachidonic acid metabolism	Arachidonic acid metabolism
Arginine and Proline Metabolism	Arginine and proline metabolism
beta-Alanine metabolism	beta-Alanine metabolism
Bile acid biosynthesis	Bile acid biosynthesis
Squalene and cholesterol biosynthesis	Biosynthesis of steroids
Butanoate metabolism	Butanoate metabolism
C21-steroid hormone biosynthesis and metabolism	C21-Steroid hormone metabolism
Proteoglycan biosynthesis	Chondroitin sulfate biosynthesis
TCA cycle	Citrate cycle (TCA cycle)
Methionine and cysteine metabolism	Cysteine metabolism
Glycerophospholipid metabolism	Ether lipid metabolism
Vitamin B9 (folate) metabolism	Folate biosynthesis
Fructose and mannose metabolism	Fructose and mannose metabolism
Galactose metabolism	Galactose metabolism
Glutamate metabolism	Glutamate metabolism
Glycerophospholipid metabolism	Glycerophospholipid metabolism
Glycolysis and Gluconeogenesis	Glycolysis / Gluconeogenesis
Glycosphingolipid biosynthesis - ganglioseries	Glycosphingolipid biosynthesis - ganglioseries
Glycosphingolipid biosynthesis - globoseries	Glycosphingolipid biosynthesis - globoseries
Glycosphingolipid biosynthesis - lactoseries	Glycosphingolipid biosynthesis - lactoseries
Glycosphingolipid biosynthesis - neolactoseries	Glycosphingolipid biosynthesis - neolactoseries
Phosphatidylinositol phosphate metabolism	Glycosylphosphatidylinositol(GPI)-anchor biosynthesis
Histidine metabolism	Histidine metabolism
Phosphatidylinositol phosphate metabolism	Inositol phosphate metabolism
Linoleate metabolism	Linoleic acid metabolism
Lysine metabolism	Lysine degradation
Xenobiotics metabolism	Metabolism of xenobiotics by cytochrome P450
Methionine and cysteine metabolism	Methionine metabolism
N-Glycan biosynthesis	N-Glycan biosynthesis
Vitamin B3 (nicotinate and nicotinamide) metabolism	Nicotinate and nicotinamide metabolism
O-Glycan biosynthesis	O-Glycan biosynthesis
Vitamin B9 (folate) metabolism	One carbon pool by folate
Pentose phosphate pathway	Pentose phosphate pathway
Tyrosine metabolism	Phenylalanine metabolism

Continued on next page

zebrafish pathway	human pathway
Tyrosine metabolism	Phenylalanine, tyrosine and tryptophan biosynthesis
Porphyrin metabolism	Porphyrin and chlorophyll metabolism
Propanoate metabolism	Propanoate metabolism
Purine metabolism	Purine metabolism
Pyrimidine metabolism	Pyrimidine metabolism
Glycolysis and Gluconeogenesis	Pyruvate metabolism
TCA cycle	Reductive carboxylate cycle (CO2 fixation)
Vitamin A (retinol) metabolism	Retinol metabolism
Selenoamino acid metabolism	Selenoamino acid metabolism
Tyrosine metabolism	Styrene degradation
Valine, leucine and isoleucine degradation	Synthesis and degradation of ketone bodies
Squalene and cholesterol biosynthesis	Terpenoid biosynthesis
Tryptophan metabolism	Tryptophan metabolism
Tyrosine metabolism	Tyrosine metabolism
Urea cycle/amino group metabolism	Urea cycle and metabolism of amino groups
Valine, leucine and isoleucine degradation	Valine, leucine and isoleucine degradation

4 Manual curation

The above procedure produced the major body of MetaFishNet version 1.8, which adhered to the pathway organization in reference models as much as possible. To improve the model quality, additional rounds of manual curation, with the help of modularity analysis, were carried out to update MetaFishNet to version 1.9. The major manual changes of pathways are listed below.

New pathways identified by module-finding and manual inspection:

sialic acid metabolism

dynorphin metabolism

electron transport chain

parathion degradation

hexose phosphorylation

Pathways that are augmented by newly identified modules:

Vitamin B1 (thiamin) metabolism

Glycosphingolipid biosynthesis - globoseries

Glycosphingolipid metabolism

Urea cycle/amino group metabolism

Methionine and cysteine metabolism

Aminosugars metabolism

C21-steroid hormone biosynthesis and metabolism

Starch and Sucrose Metabolism

Pentose phosphate pathway

Pathway merging:

Oxidative Phosphorylation to electron transport chain

Endohydrolysis of 1,4-alpha-D-glucosidic linkages in polysaccharides by alpha-amylase to Starch and Sucrose Metabolism

Pathways that were dismantled to isolated reactions because of too few reactions:

Diterpenoid biosynthesis

D-arg and D-orn metabolism

Caprolactam degradation

Benzoate degradation via hydroxylation

Aminophosphonate metabolism

Stilbene, coumarine and lignin biosynthesis

Naphthalene and anthracene degradation

5 Databases and web service

All gene sequences (cDNA, UTRs included) of zebrafish, medaka, Takifugu, Tetraodon and stickleback were exported from Ensembl database via its BioMart feature. Their human homologs and zebrafish GO annotation were also retrieved from Ensembl, then reconstructed into local MySQL databases. A local copy of complete Gene Ontology database (seqdblite) is maintained. The core data for MetaFishNet were compiled into a new MySQL database (as in Figure 4).

The MetaFishNet core database defines the relationships among genes, enzymes, compounds, reactions and pathways. Primary gene IDs were adopted from Ensembl. This MetaFishNet database also includes zebrafish gene IDs from GenBank and ZFIN, so that users can look up genes by these ID systems. However, fish genomics is still evolving and most gene identifications will have to be established via sequence comparison by *SeaSpider*. Besides the relational databases, three sequence databases are used with SeaSpider and BLAST (Figure 2): zebrafish sequences, generic sequences associated with Gene Ontologies and MetaFishNet sequences, which consist of all metabolic genes from five fish species used in the construction.

We use Google App Engine (GAE) to build our project website [35]. GAE provides a free (within quota) and stable platform, which eliminates logistic costs of maintaining the website. The web development

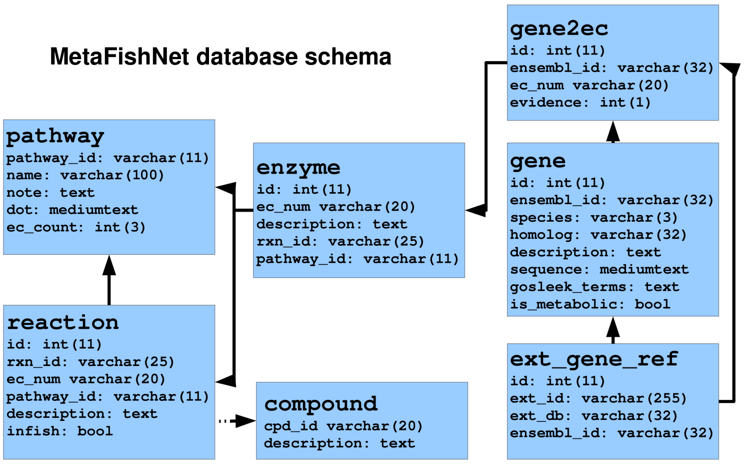

Figure 4: Database schema for MetaFishNet. The linkage between "compound" and "reaction" is not directly through attribute matching. A simple text parsing of reaction.description makes the connection. This trick saves storage space and improves database performance.

framework of GAE (similar to the popular Django framework) is state of the art, enabling rapid development and deployment. We ported our database to Google's datastore to support this website (Figure 5). However, the choice of GAE also limits functionalities of the site. Extensive use of CPU is disallowed and regular programs cannot be installed. This prevents the deployment of FishEye and SeaSpider on the project site, though both programs can be downloaded and run locally.

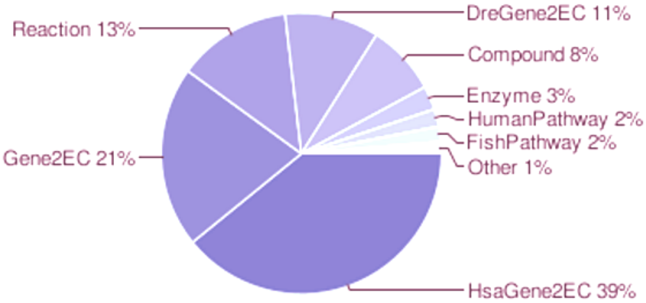

Figure 5: Data stored on Google App Engine, showing size percentages of tables/entities. GAE storage is a graphic/object database, not relational.
